# The Exploitation of Liposomes in the Inhibition of Autophagy to Defeat Drug Resistance

**DOI:** 10.3389/fphar.2020.00787

**Published:** 2020-05-29

**Authors:** Maria Condello, Giovanna Mancini, Stefania Meschini

**Affiliations:** ^1^National Center for Drug Research and Evaluation, National Institute of Health, Rome, Italy; ^2^Institute for Biological Systems, National Research Council, Rome, Italy

**Keywords:** cancer, drug resistance, chemotherapy, autophagy inhibitors, liposomes

## Abstract

Autophagy is a mechanism involved in many human diseases and in cancers can have a cytotoxic/cytostatic or protective action, being in the latter case involved in multidrug resistance. Understanding which of these roles autophagy has in cancer is thus fundamental for therapeutical decisions because it permits to optimize the therapeutical approach by activating or inhibiting autophagy according to the progression of the disease. However, a serious drawback of cancer treatment is often the scarce availability of drugs and autophagy modulators at the sites of interest. In the recent years, several nanocarriers have been developed and investigated to improve the solubility, bioavailability, controlled release of therapeutics and increase their cytotoxic effect on cancer cell. Here we have reviewed only liposomes as carriers of chemotherapeutics and autophagy inhibitors because they have low toxicity and immunogenicity and they are biodegradable and versatile. In this review after the analysis of the dual role of autophagy, of the main autophagic pathways, and of the role of autophagy in multidrug resistance, we will focus on the most effective liposomal formulations, thus highlighting the great potential of these targeting systems to defeat cancer diseases.

## Introduction

Our work is focused on the pro-tumorigenic role of autophagy in cancer, and analyzes recent acquisitions on the connection between multidrug resistance (MDR) and autophagy and the possibility of defeating MDR by specific autophagy inhibitors, whose bioavailability at the target site can be greatly improved by using liposomes as drug nanocarriers. Accordingly, the review is organized in four paragraphs that analyze respectively: 1) the role and mechanism of autophagy in cancer; 2) the drug resistance mechanisms involved in the survival of cancer cells and its connection with autophagy; 3) the main autophagic modulators and their use in combined therapies to defeat resistance over cancer treatment and, finally, 4) the use of liposomes for an effective and simultaneous transport of chemotherapeutics and inhibitors of autophagy to control pharmacokinetics and targeting, thus reducing adverse effects of drugs.

## Autophagy In Cancer

**Figure 1:**
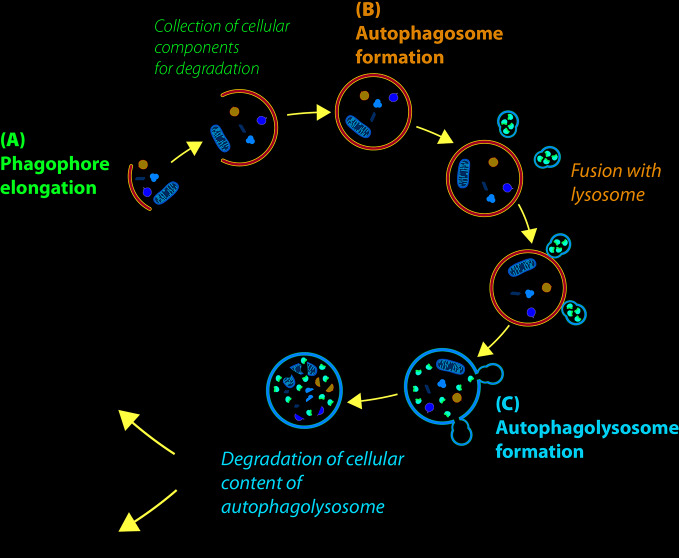
Description of cellular autophagic process. **(A)** After the stress signal, a small double membrane, called phagophore, grows to incorporate the cellular components that to need to be degraded, to form autophagosome **(B)**. **(C)** Autophagosome fuses with lysosome to form autophagolysosomes, where hydrolytic enzymes degrade cellular components. Sometimes, the material is recycled to supply energy (autophagy-mediated cell survival); otherwise, when autophagy levels are very high, cell dies by autophagic cell death (ACD).

Autophagy is a highly conserved cell process, in which cytoplasmic materials (as defective organelles or useless molecules or structures such as misfolded proteins, excessive peroxisomes, ribosomes and invading pathogens) are degraded and recycled to maintain energy homeostasis. The process begins with the formation of an isolated membrane, called phagophore, a lipid double-membrane that envelops cellular materials and recruits autophagy-related proteins to induce autophagy (**Figure 1A**). The phagophore expands to form an autophagosome, a double-membrane vacuole, which contains part of cytoplasmic components (**Figure 1B**). The autophagosome size and number are important for the selectivity of the cargo and can be decisive for the regulation of the autophagic flow. Autophagosome later moves to lysosome using microtubules for fusion and formation of autophagolysosome (**Figure 1C**) (Wei et al., 2018). The acid-dependent hydrolases present in the lysosome degrade the content of autophagosomes, and macromolecules are exported to the cytosol to be reused in the cell.

The autophagic basal levels ensure the quality of cellular components and the maintenance of cellular homeostasis through the regular replacement of long-lasting proteins, the elimination of protein aggregates and damaged organelles. The cytoplasmic organelles that need to be limited can be identified and eliminated through a mechanism called “selective autophagy” (Zaffagnini and Martens, 2016). Under various conditions, different selective autophagy depending on the organelle, to be eliminated, are described: mytophagy (for mitochondria), ribophagy (for ribosomes), reticulophagy (for endoplasmic reticulum), lysophagy (for lysosomes), pexophagy (for peroxisomes), lipophagy (for lipid drops), glycophagy (for glycogen), aggrephagy (misfolded proteins), and xenophagy (infected pathogens) (Stolz et al., 2014).

Autophagy is a dynamic and multiphase process; tests of genetic screening on the yeast *Saccharomyces cerevisiae* have identified 30 genes, whose protein products are able to control autophagic phases. They precisely called ATG genes (AuTophaGy related genes) (Tsukada and Ohsumi, 1993). The sequences of ATG genes are homologous in higher eukaryotes, suggesting that the molecular mechanism of autophagy is highly conserved in evolutionary scale. Moreover, other proteins, belonging to kinases class, regulate the autophagic process in a highly specific way (Klionsky et al., 2012).

**Figure 2:**
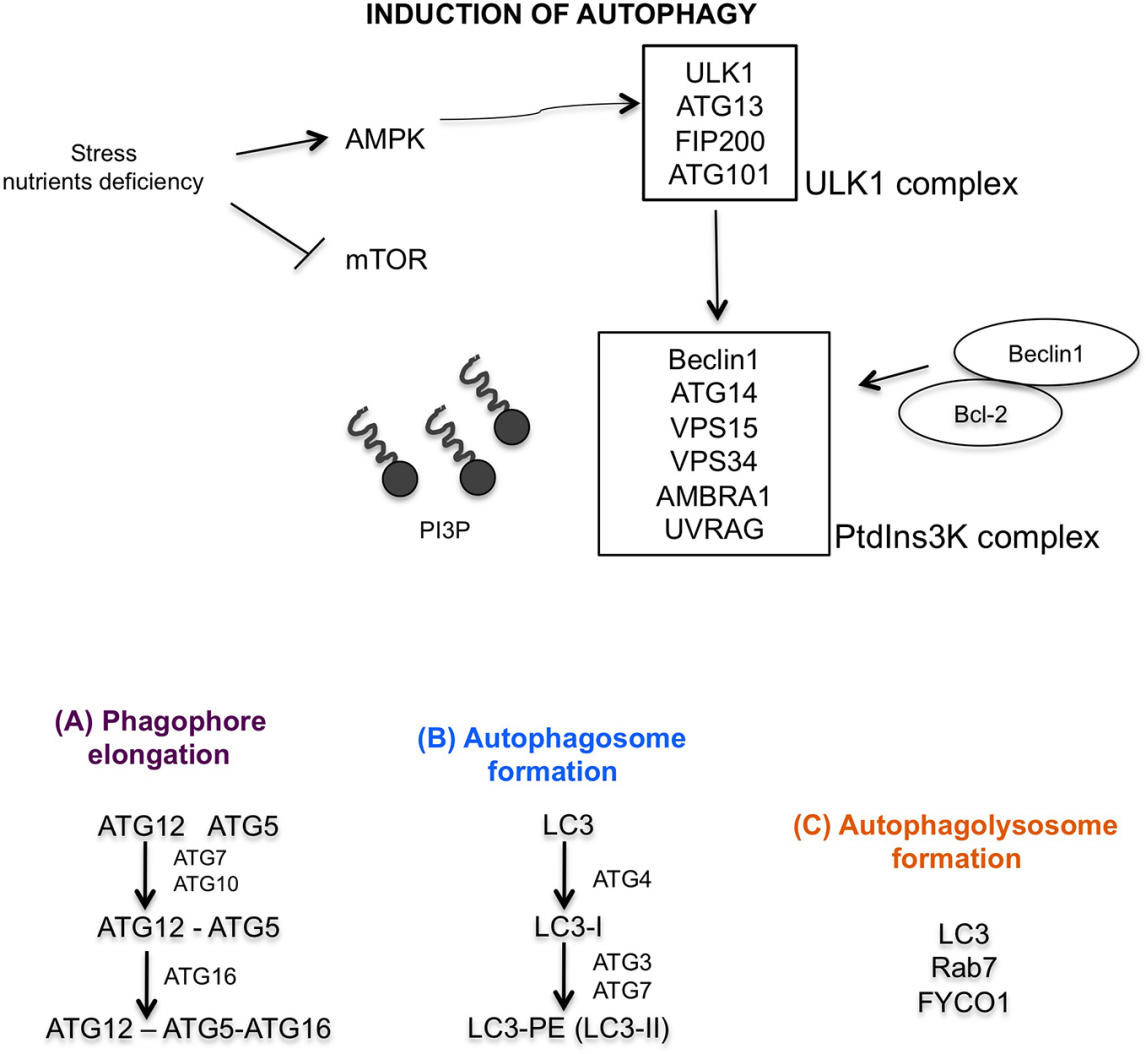
Graphic illustration of molecular autophagic pathway. Induction of autophagy characterized by mTOR inhibition, activation of AMPK, ULK1 and PtdIns3K complexes. **(A)** Regulation of phagophore elongation by ATG12-ATG5-ATG16 complex. **(B)** Autophagosome formation mediated by LC3 maturation, and finally **(C)** autophagolysosome formation mediated by LC3, Rab7, and FYCO1 proteins.

The central modulator of autophagy regulation is the mammalian target of rapamycin (mTOR) which responds to microenvironment intracellular changes such as deprivation of amino acids and glucose, and therapeutic treatments, irradiation, hypoxia (Stephan et al., 2009). In physiological condition, mTOR is active and inhibits autophagy and protein degradation. Under induction of cellular stress, mTOR is inactive, dephosphorylates ULK1 complex (that includes ULK1, ATG13, Focal adhesion kinase family interacting protein of 200 kDa (FIP200) and ATG101 protein). ULK1 complex dissociates from mTOR complex, and AMPK phosphorylated ULK1 complex, triggering autophagy (Wong et al., 2013). The activation of phosphatidylinositol 3-kinase (PtdIns3K) complex (formed by Beclin1, ATG14, vacuolar protein sorting (VPS15), VPS34, activating molecule in BECN1 regulated autophagy protein 1 (AMBRA1), and ultraviolet irradiation resistance-associated gene (UVRAG)) follows (Russell et al., 2013). This activation is further regulated by Beclin1–Bcl-2-complex (Pattingre et al., 2005). The induction of PtdIns3K complex generates the lipid phosphatidylinositol-3-phosphate (PI3P), which recruits other proteins essential for phagophore formation (**Figure 2**). In particular ATG12–ATG5–ATG16 complex and ATG9, ATG2, and WIPI 1/2 proteins are involved for elongation of phagophore (**Figure 2A**) (Hurley and Young, 2017). The second conjugation complex is ATG8 protein, also known as microtubule-associated protein 1 light chain 3 (MAP1-LC3 or LC3) (Kirisako et al., 2000). This protein is inactive form free in the cytosol; the C-terminal end is cleaved by the ATG4 protease, thus producing a new form, called LC3-I, that is subsequently conjugated to phosphatidylethanolamine (PE) by ATG3/ATG7 system (Satoo et al., 2009). After conjunction, LC3-I is converted to LC3-II form, which is exposed on external side of mature autophagosome (**Figure 2B**) (Ichimura et al., 2000). Mature autophagosome travels along the microtubule towards the lysosome. This transport is mediated by an adaptor protein complex formed by LC3, Rab7, and FYCO1 (**Figure 2C**) (Pankiv et al., 2010). Finally, after formation of autophagolysosome, LC3-II protein is internalized, PE residue is detached by hydrolytic lysosomal enzymes and the protein is released in the cytoplasm with consequent decreased expression (Tanida et al., 2008).

Because autophagy is an important cell quality control process, its dysregulation is involved in several diseases, as metabolic disorders, neurodegenerative diseases, autoimmune alterations and cancer (Condello et al., 2019).

**Figure 3:**
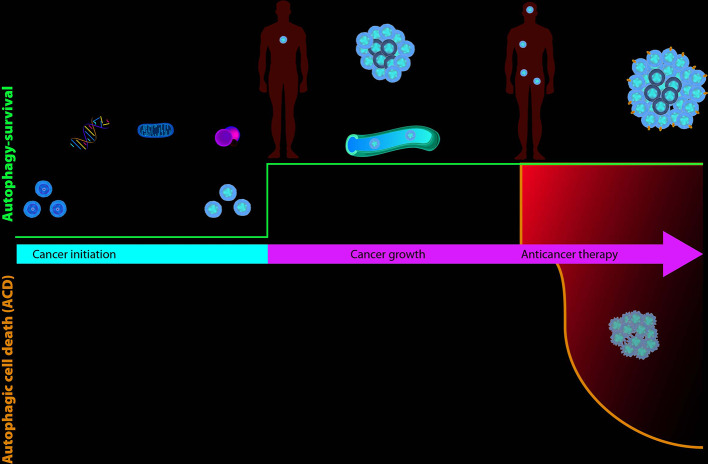
The role of autophagy, as survival or death mechanisms, in cancer. If autophagy is downregulated, healthy cells can be damaged due to DNA or mitochondria alterations or the production of reactive oxygen species (ROS) and turn into cancer cells. Upregulation of autophagy allows cancer cells to survive hypoxia and hypoglycemia of the microenvironment and promotes tumor growth and the spread of metastases. During cancer therapy, autophagy allows cancer cells to survive the cytotoxic effect of drugs and promote resistance (MDR), or alternatively, prolonged activation of autophagosomal pathway leads to autophagic cell death (ACD).

Numerous alterations in of the expression of autophagic genes have been reported in several types of cancer such as pancreatic, lung, bladder and breast cancer; in fact, the monoallelic deletion of genes such as ATG5, ATG6, ATG7 and the total loss of ATG4 have been linked to the risk of induction of malignancies (Mariño et al., 2007; Takamura et al., 2011). However, the role of autophagy in the various stages of cancer progression is contradictory (**Figure 3**) (Singh et al., 2018). Autophagy has a tumor promoting role, favoring cancer growth under hypoxia or nutrient limitation, avoiding cell apoptotic death, and maintaining dormancy. On the other side, autophagy has a tumor-suppressive role maintaining genome integrity and preventing metastases.

Reduced levels of autophagy are characteristic of the early stage of cancer. This situation leads to the accumulation of oncogenes and reactive oxygen species (ROS) (Poillet-Perez et al., 2015). Instead, autophagy is upregulated when tumor growth is activated; in this way an adequate supply of energy is guaranteed and the cancer cell is able to survive stress, hypoxia and metastasis (Mowers et al., 2017). During anticancer therapy, autophagy is increased, cells survive to cytotoxic effect of drugs and autophagy induces therapy resistance (Li et al., 2017).

In recent studies, the role of the autophagic component in the survival mechanism of cancer stem cells (CSCs) has been shown. CSCs live in hypoxic, nutrient-poor and acidic environment conditions, are highly responsive to these stimuli, and are able to regenerate themselves to induce the formation of metastases and resistance to drug therapy. CSCs have higher basal autophagy than non-cancerous SCs (Boya et al., 2018). However, the problem of being able to convert CSC to non-CSC *in vivo* by inhibiting systemic autophagy has not yet been resolved. Some authors have actually observed that the depletion of autophagy decreased the viability of chronic myeloid leukemia CD34+ progenitor cells, whereas its inhibition in hematopoietic stem cells (HSC) has favored the development of hematopoietic diseases (Auberger and Puissant, 2017; Levine and Kroemer, 2019). On the other hand, after treatment with synthetic or natural compounds, there are some evidences that when the autophagosomal/lysosomal pathway is over activated for a prolonged time the autophagic cell death (ACD, type II cell death) can occur (Liao et al., 2019; Linder and Kögel, 2019). Arsenic trioxide (ATO) has been proved to induce ACD in several cancer cells. It is an interesting compound capable of crossing the blood–brain-barrier. ATO can be used for targeting the expression of stemness marker genes inhibiting the Hedgehog signaling pathway and Notch signaling pathways and inducing cell death in brain tumors. Furthermore, the combined effect of ATO and gossypol (AT-101, Gos), a natural compound, has been shown to induce cell death diminishing the self-renewal capacity of tumor sphere lines (Linder et al., 2019). A recent article of Cordani and Somoza (2019) highlighted that the use of metal-based nanoparticles has an opposite autophagic role on cell fate. Metal-based nanoparticles can modulate autophagy by inducing cell death through multiple mechanisms, such as induction of ER stress leading to oxidative stress, mitophagy, mitochondrial and lysosome damage in cancer cells and pro-survival autophagy in cancer and in normal cells (Cordani and Somoza, 2019). Autophagy, as previously mentioned, can act as tumor promoter or suppressor, its role strongly depends on the context of the tumor. The presence of hypoxia or the lack of nutrients can trigger the autophagic mechanism, which can facilitate the tumor adaptation to different stress thus inducing tumor progression. Therefore, it is very important to know the context of the disease in its complexity, indeed the inhibition or activation of autophagy might improve the therapeutic response. Based on the dual role of autophagy in cancer and on the coexistence of multiple mechanisms (autophagy/apoptosis) within a tumoral mass, it is crucial to study compounds able to modulate autophagic process. In recent years, many compounds, that induce or inhibit autophagy, have been identified for pharmacological intervention.

## Multidrug Resistance In Cancer

Although cancer treatment is constantly evolving, yet it is third leading cause of death in the worldwide (Bray et al., 2018). The main reason of this high mortality is the lack of effective treatments and the onset of the resistance. The resistance can be intrinsic or acquired. Intrinsic resistance is due to genetic alterations, the tumor become resistant to drug before the treatment. Up to a few years ago, intrinsic resistance was considered the main mechanism of resistance. Acquired resistance, induced by drug administration, is also an important responsible of treatment failure in cancer patients. When tumor cells become resistant to pharmacologically and structurally distinct class of drugs, chemoresistance is defined multidrug resistance (MDR) (Baguley, 2010).

One of the most important mechanisms underlying MDR is the overexpression of adenosine triphosphate (ATP)-binding cassette (ABC) superfamily of transporters (Chen et al., 2016). Among the 48 ABC transporters identified in humans, those localized on plasma membrane increase the efflux of anticancer drugs and reduce their intracellular accumulation using ATP energy. The major ABC transporters involved in MDR are P-glycoprotein (P-gp/ABCB1), multidrug resistance-associated protein 2 (MRP2/ABCC2), and breast cancer resistance protein (BCRP/ABCG2) (Locher, 2016).

**Figure 4:**
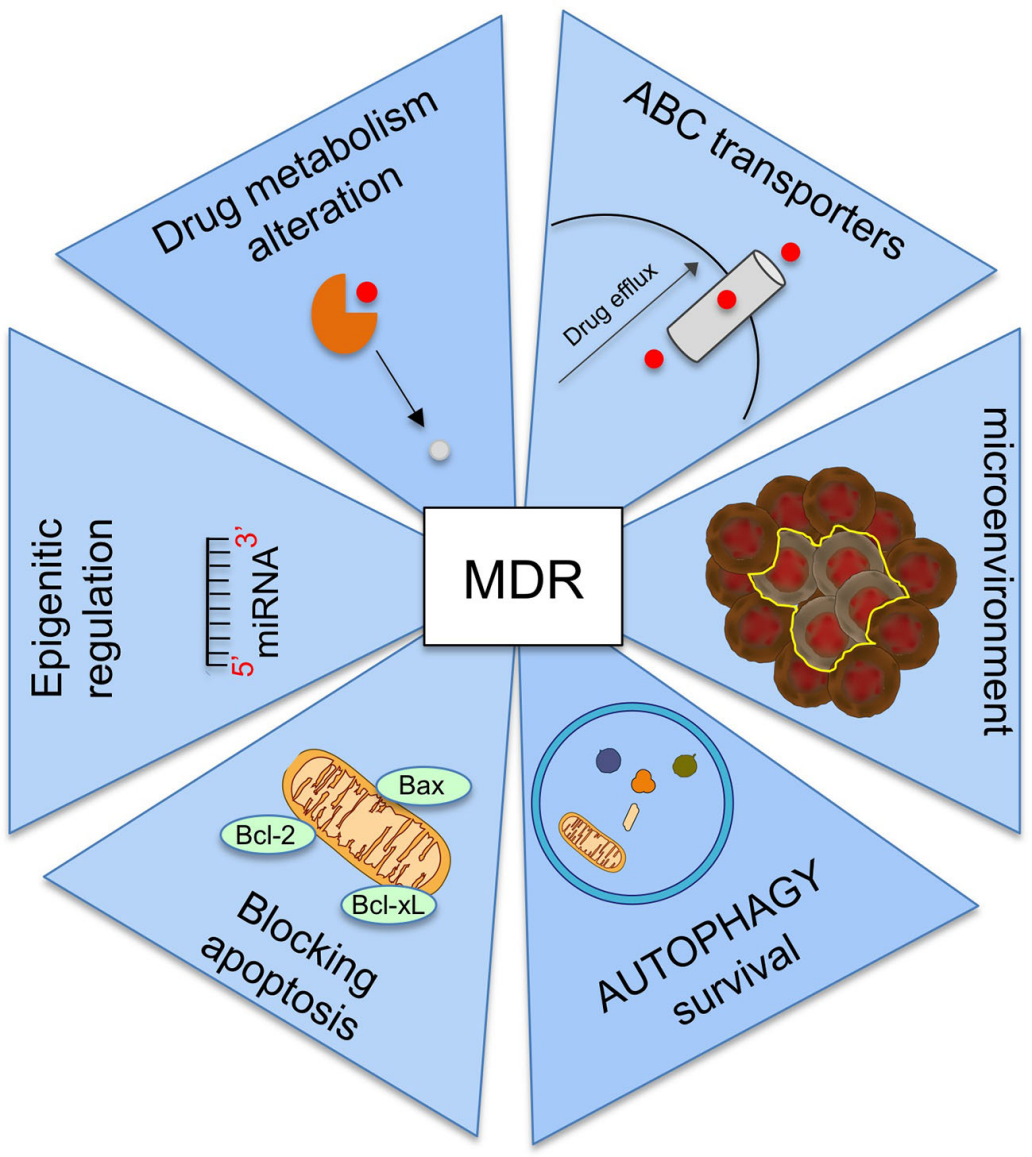
Summary of the main mechanisms involved in the onset of multi-drug resistance (MDR).

Other mechanisms involving in MDR are (**Figure 4**):

− the alteration of drug metabolism by activation of detoxifying systems as glutathione-S-transferase and cytochrome P450 enzymes (Joyce et al., 2015);
− the block of apoptotic signaling pathway by upregulation of antiapoptotic proteins, downregulation of proapoptotic proteins, and by induction p53 pathway mutations (Fanourakis et al., 2018);
− the increasing the adaptability by epigenetic regulation and microRNA regulation (Wengong et al., 2019);
− mutations in drug target (Camidge et al., 2014);
− the change of tumor microenvironment (Mowers et al., 2018).

Numerous evidences suggested the role of tumor microenvironment in cancer MDR (Al-Akra et al., 2019). The tumor microenvironment is a dynamic network of cancer cells, stromal cells and extracellular matrix characterized by deficiency of oxygen (hypoxia), nutrients and glucose (hypoglycemia), as well as by a low pH. Hypoxic conditions lead to an inadequate number of vessels within the tumor mass, resulting in the reduction of the nutrients and cytotoxic drugs to the tumor cells with reduced drug efficacy.

The main key regulator of the tumor microenvironment to control MDR is alpha subunit of hypoxia-inducible factor1 (HIF1-α). When oxygen supplies are low, HIF1-α expression up-regulates both the transport of glucose by GLUT1 across the plasma membrane and the genes involving in glycolysis. Consequently, this regulation mediates cell metabolism switch from mitochondrial oxidative phosphorylation to glycolysis (Hayashi et al., 2004). As result of enhanced glycolysis, ATP levels increase and can directly influence the activity of ABC transporters, which activate drug efflux and promote chemoresistance (Bhattacharya et al., 2016). HIF1-α levels up-regulate expression of Vascular-Endothelial Growth Factor (VEGF), that induces expression of anti-apoptotic proteins as Bcl-2, and increases MRP1 expression promoting MDR. Moreover, another target of HIF-α is nuclear factor (erythroid-derived 2)-like 2 (Nrf-2), which interacts with the autophagy pathway leading to drug therapy resistance by autophagy. Nrf-2 can also increase the expression of ABC transporters as ABCB1, ABCG2, and ABCC2 and regulate the enzymes involved in phase II drug metabolism (Shen and Kong, 2009).

Hypoglycemia creates an imbalance between oxidant and anti-oxidant products, which results in amplified levels of ROS. Oxidative stress activates signal transduction pathways that increase the expression of anti-apoptotic and anti-oxidant molecules (catalase and superoxide dismutase), promoting drug resistance and cell survival. In particular, hypoxia modulates the expression of proteins regulating apoptosis. The expression of some pro-apoptotic Bcl-2 family, including Bax, Bad and Bid, is decreased under hypoxia, whereas the expression of anti-apoptotic Bcl-2 and Bcl-xL is increased (Matsuura et al., 2016). Since some anticancer drugs induce cell death by apoptosis, the down regulation of this mechanism can suppress treatment efficacy. Clinical trials showed that hypoxia induce resistance to chemotherapy with anthracycline drugs due to overexpression of anti-apoptotic Bcl-2.

## MDR and Autophagy

In the tumor microenvironment the autophagy mechanism plays an interesting role, it is able to generate MDR. Hypoxic stress of tumor microenvironment induces the expression of BH3 domains of B-cell lymphoma 2 (Bcl-2)/adenovirus E1B 19-kDa protein-interacting protein 3 (BNIP3), which is involved in dissociation of Bcl-2–Beclin1 complex. As result, displaced Beclin1 can trigger autophagy (Bellot et al., 2009). In poorly oxygenated regions of the bone marrow after the onset of acute myeloid leukemia and in the hypoxic areas of solid tumors, autophagy regulatory pathway has an important role during adaptation of cancer cells to hypoxic stress (Rouschop et al., 2010). In a panel of cancer cell lines, cell death increased upon knockdown of Beclin1 or ATG7 genes (Tan et al., 2016), suggesting a role of autophagy in cell survival within hypoxic regions. The key molecule is HIF1-α, which activates autophagy by regulating Beclin1 (Wu et al., 2015).

Even in the metastatic and anoikosis regions the autophagy is crucial for cancer cell survival. Anoikis is a programmed cell death induced when tumor cells detached from the substrate due to loss of contact with the extracellular matrix. This mechanism could prevent the dissemination of cancer cells to other parts of the body. In fact, cells migrate from the tumor, travel through the blood or lymphatic system to initiate a new tumor mass in other organs (metastases). Cancer cells acquire anoikis resistance to survive cell death due to loss of contact with the extracellular matrix. As shown in metastatic hepatocellular carcinoma, anoikis resistance is due to autophagic pathway induction (Fu et al., 2018). Moreover, the level of LC3-II is significantly higher in metastases of breast and liver cancer and melanoma than in primary tumors (Lazova et al., 2012).

In addition, many studies demonstrated that autophagy is induced in tumors after many therapeutic approaches, including irradiation, conventional therapies and targeted therapies (Levy et al., 2017). Consequently, the autophagic process protects tumor cells recycling intracellular components and maintaining functional pool of mitochondria. Therefore, autophagy/mitophagy promote tumor growth and they contribute to drug resistance (Li et al., 2017). Many studies demonstrated that autophagy triggered by chemotherapy facilitates the resistance of cancer cells to paclitaxel, tamoxifen, epirubicin or trastuzumab (Vazquez-Martin et al., 2009; Ajabnoor et al., 2012). For example, autophagy is induced by cisplatin treatment, by activation of DRAM1, a p53-mediated regulator (Crighton et al., 2006). In the event of imatinib treatment of gastrointestinal stromal tumor cells, induction of autophagy has been observed and with Chloroquine treatment it is inhibited in favor of apoptosis (Gupta et al., 2010). In different types of tumor (breast, lung and cervical) the irradiation treatment induced an increase in autophagy, if instead inhibition of autophagy was induced, a reduction in the formation of colonies in clonogenic tests was observed (Apel et al., 2008). The induction of autophagy-mediated cancer resistance mechanism after therapeutic approaches is attributed to reduced mTOR activity (Amaravadi et al., 2011), or to turnover of FOXO3A that reduced apoptosis by preventing FOXO3A-dependent induction of Puma, a BH3 proapoptotic protein (Fitzwalter et al., 2018). In other situations, the increased production of ROS after mitochondrial damage or ER stress may explain the occurrence of autophagy *via* activation of FOXO, ATG5 and LC3 and other autophagic proteins (Ranganathan et al., 2006).

Further it has been suggested that autophagy may also promote drug resistance by favoring selection of cancer stem cells phenotype in breast cancer and glioblastoma (Smith and Macleod, 2019).

CSCs are a small population of cancer cells that are the cause of tumor heterogeneity. They are resistant to conventional therapies and are characterized by high levels of autophagy, thanks to which they maintain pluripotency, supply nutrients and oxygen to the microenvironment, they also have a high metastatic potential, regulate invasion and migration, induce resistance to drugs and allow cancer cells to escape immune surveillance (Nazio et al., 2019). In particular, CSCs depend on mitophagy to degrade damaged mitochondria, thus maintaining a low ROS level and preventing the activation of programmed cell death. In hepatic CSCs the activation of Pink1-dependent mitophagy (p53, Pink1, NANOG) promotes cell proliferation (Liu et al., 2017).

A connection between CSCs, autophagy and drug resistance has been noted in many cancers such as melanoma, leukemia, brain, pancreas, colon and breast (Abdullah and Chow, 2013). As revealed by several experimental approaches, the sensitivity of CSCs increased when the cells were treated with the combination of cytotoxic drugs and autophagy inhibitors. Indeed, in glioblastoma the simultaneous treatment of Bevacizumab and Temozolomide with Chloroquine, stem glioblastoma cells became more sensitive and their survival was reduced (Huang et al., 2018).

At the end of this rapid resume on MDR it is important to highlight the double role that autophagy plays in this mechanism; it is able to protect cancer cells from drug therapy but in some cases, when the path of apoptosis does not work, it can kill the cancer cell. Therefore, the understanding the contribution of autophagy to cancer drug resistance is crucial to develop novel antineoplastic therapies, based on combination of autophagy inhibitors with cytotoxic drugs to sensitize refractory cancers.

## New Autophagy Inhibitors For Cancer Treatment

As previously described, autophagy has a dual role in inducing death or promoting the survival of cancer cells. Autophagy in its pro-tumor function, increases resistance to apoptosis, prevents the induction of tumor suppressors and maintains the tumor metabolism through the recycling of nutrients. Its modulation in association with chemotherapeutic agents has been considered a promising therapeutic option. The main inhibitors of the autophagic mechanism developed to date work by blocking the nucleation and extension of the phagophore or by blocking the endosomal/lysosomal acidification process.

In this section we have highlighted some autophagy inhibitors that in specific tumor context have demonstrated a mass regression. Melanoma is a very aggressive tumor, has a primary skin site and multiple metastases that explain the morbidity and mortality in the population. Recently, verteporfin, a benzoporphyrin derivative, has been shown to have an anti-tumor effect on melanoma cells *in vitro*, in a preclinical model of transgenic mouse (Lu et al., 2019) and in the clinical treatment of macular degeneration. Verteporfin action consists in inhibiting the formation of autophagosomes by depriving the cells of glucose and serum, by blocking the p62 oligomerization, a protein useful for the sequestration of ubiquinated targets into autophagosomes. It induces apoptosis and inhibits cell proliferation, angiogenesis and migration (Lui et al., 2019).

On the market there are two drugs, inhibiting the autophagic process for the treatment of cancer, are chloroquine (CQ) and hydrocholoroquine (HCQ), a less toxic metabolite and used to treat rheumatic diseases, such as rheumatoid arthritis and systemic lupus erythematosus. CQ, a drug used to treat malaria caused by several Plasmodium strains, unfortunately has many side effects if used for prolonged times. The adverse effects of CQ are associated with cardiovascular side effects, particularly hypotension and electrocardiographic QT interval prolongation. CQ and HCQ, are weak bases which, once into the lysosomes, are protonated thus inhibiting the activity of the lysosomal enzymes. CQ and HCQ are the only autophagy inhibitors approved in the phases I and II clinical trials, however, adverse effects, have been observed in prolonged treatments in combination with chemo or radiotherapy (Xu et al., 2018). Extensive research was needed due to the observed side effects. A meta-analysis study in which 249 patients with solid and non-solid tumors were analyzed and compared showed an improvement in overall survival at one year, although at various levels depending on the tumor and the drugs combination analyzed (Xu et al., 2018). The different response observed in these compounds from one tumor to another is due to the reason why autophagy is hyperactivated in many tumors, but not all are sensitive to autophagic inhibitors.

A dimeric analogue of CQ with the potential to accumulate in acidic organelles is commercially known as LYs05. It shows an inhibition of autophagy higher than HCQ. In C8161 melanoma cells, it induces p62 accumulation and increases the ratio of LC3-II to LC3-I in a concentration-dependent manner. The combined treatment of Lys05 and ionizing radiation in lung cancer cells decreased cell survival by inhibiting autophagy (Cechakova et al., 2019).

Afterwards the researchers have focused on developing of another analogous dimeric CQ inhibitor, DC661, which is a potent inhibitor of autophagy and cancer cell proliferation. DQ661 is a dimeric quinacrine, which has proven effective on an immunocompetent mouse model of cancer overcoming the gemcitabine resistance. It induces alteration in multiple lysosomal functions as macropinocytosis and mTORC1 activity by selectively targeting protein-palmitoyl thioesterase 1 (PPT1), a key regulator of palmitoylation within the lysosome (Nicastri et al., 2018). In the class of antimalarial quinolinic agents to which chloroquine also belongs, another interesting compound is mefloquine (MQ) considered a lysosomotropic agent and an autophagy inhibitor used for various tumor pathologies. In the work of Sharma et al. (2012) MQ was used as an inhibitor of autophagy against breast cancer cells, in this case MQ inhibited autophagy in the formation phase of autophagosomes in hormone positive and triple negative breast cells. However, long-term use of MQ has been reported to have neurological and psychiatric adverse effects in some patients. It was also recently shown to induce apoptosis by sensitizing patient-derived myeloid leukemia cells to tyrosine kinase inhibitors (Lam Yi et al., 2019). The macrolide antibiotic bafilomycin A1 was isolated from *Streptomyces gresius* and has been shown to be a potent inhibitor of the Vacuolar H^+^ATPase which controls pH in the lysosome (V-ATPase) (Bowman et al., 1988). Its mechanism of action is the inhibition of lysosomal acidification by averting the passage of protons in the lysosomal lumen. Bafilomycin A1 inhibits autophagosome–lysosome fusion by blocking V-ATPase pump activity and disrupting Ca^2+^ gradient involved in this process (Mauvezin and Neufeld, 2015). Protease inhibitors named, pepstatin A (aspartyl protease; cathepsin D and E), leupeptin and E64d (cysteine proteases, cathepsin B, H and L) can also inhibit the lysosomal degradation (Tanida et al., 2005). Uncoordinated 51-like kinase 1 (ULK1) dysregulation has been found in many human cancers in particular in clear cell renal cell carcinoma (ccRCC) (Lu et al., 2018). It is known, in fact, that ULK1 regulator, essential for starting autophagy (Wong et al., 2013), is very often related to drug resistance and poor prognosis. SBI-0206965, an inhibitor of ULK1 activity has been shown to inhibit autophagy and to induce apoptosis in renal carcinoma cells, in neuroblastoma cells and in non-small cell lung cancer (Martin et al., 2018). Prostate cancer (PCa) is a serious problem in the population, due to the development of drug resistance after surgery. The epidermal growth factor receptor (EGFR) is known as a therapeutic target for the treatment of numerous human carcinomas; it has recently been discovered that it has an important role in the progression of resistant PCa. Spautin-1, a Beclin1 inhibitor, significantly suppressed the growth of PCa by arresting cell cycle progression and triggering apoptosis (Lu et al., 2018). Spautin 1, can also reduce the apoptosis block induced by low levels of inflammatory cytokine, IL-17 (Xue et al., 2019). It is known that Beclin1 promotes protective autophagy in the osteoclastogenesis by an anti-apoptotic effect.

3-methyladenine (3-MA) is one of the first used inhibitors of autophagy (Xie et al., 2016), under starving conditions it suppresses autophagy by inhibiting phosphatidylinositol 3-kinase, catalytic subunit type 3 (PI3KC3), on the other hand, with nutrients it favors autophagy by using PI3KC1 inhibition (Laha et al., 2019). Because 3-MA was shown to be effective only at high doses, other derivatives were investigated, as Wortmannin, a fungal metabolite that binds to the catalytic site of PI3Ks, and SF1126 (Gomes et al., 2019; Qin et al., 2019). SF1126 is a novel and potent dual inhibitor of phosphatidylinositol 3-kinase and bromodomain-containing protein 4 oncogenes. Suggestive activation of apoptosis and inhibition of tumor growth with xenograft HT-29 in nude mice have been revealed in colorectal cancer before (CRC) cells treated with SF1126, expressed in (CRC). VPS34 is a PI3KC3 inhibitor that forms complex for its activation with several subunits such as p150, ATG14 and Beclin1. The aurone derivative, a novel natural product, inhibitor of the VPS34 activity, upregulates p62 levels and inhibits vesicle trafficking in HeLa cells (Li et al., 2019). SAR405, highly specific VPS34 inhibitor, is one compound of the (2S)-tetrahydropyrimido-pyrimidinones series with kinase inhibitor activity, alters vesicle trafficking (late endosome-lysosome compartments) and limits autophagy. The combined use of SAR405 and everolimus has demonstrated the reduction of cell proliferation in renal cancer cells and has allowed the clinical use of PIK3C3 inhibitors in this disease (Kocaturk et al., 2019). A novel inhibitor of ATG4B activity, named NSC185058, is able to modify the volume of the autophagosomes *in vitro*, while having no effect on mTOR and PtdIns3K activities. NSC185058 has shown to have a role in the suppression of tumor growth in an osteosarcoma subcutaneous mouse model (Akin et al., 2014) and in combination with radiotherapy, in the treatment of mice with intracranial glioblastoma xenograft markedly, slows down glioblastoma growth thus inducing survival (Huang et al., 2017).

Recent and interesting work has demonstrated the inhibitory role of miR-154 against the *ATG7* autophagic gene. ATG7 gene is a direct target of miR-154. The expression of miR-154 is downregulated in bladder cancer and associated with poor survival in the patients. The overexpression of ATG7 gene induces the proliferation, migration and invasion of bladder cancer cells. MiR-154 by mitigating the expression of ATG7, may function as a tumor suppressor (Zhang et al., 2019).

## Liposomes As An Alternative Strategy Against Autophagy-Related Mdr

As discussed above the main clinical impediment in cancer treatment is the development of MDR that occurs through chemotherapy. The molecular mechanisms involved in MDR phenomenon are various: reduction of drug intake, overexpression of efflux pumps in tumor cell membrane, altered cell cycle control points, increase in drug metabolism and altered apoptotic pathway (Narayanan et al., 2020). The control of degradation processes and the recycling of cellular components during metabolic stress is attributed to the activation of autophagy. The dual role of autophagy in cancer and the choice of the best strategy to overcome MDR depends on the stage of the disease, on its progress and on the acidic microenvironment of cancer cells. The tumor microenvironment includes different cells, such as stem, stromal, endothelial cells, and innate and adaptive immune cells infiltrating tumors, all of which exploit autophagy in a different way. It is clear that targeting autophagy could be a crucial approach in the treatment of pathological conditions, however there are several impediments in the use of autophagic modulators. Actually, autophagy modulators suffer from low bioavailability due both to low solubility in aqueous media and non-target delivery, the results being a modest therapeutic efficacy. In the last years the advent of nanotechnology has opened new perspectives in many research fields and the pharmaceutical research has been engaged in finding new nanotechnology based on theurapeutical solutions, generally referred as nanomedicine. In the nanomedicine field many nanoparticles have been proposed as drug carriers and imaging tools (Bamburowicz-Klimkowska et al., 2019). Nanoparticles (NPs) have peculiar physicochemical properties, such as charge, shape, surface decoration and a high surface-to-volume ratio that make them particularly attractive to load and deliver to certain targets high number of drugs (Xin et al., 2017; Gonda et al., 2019).

Among all different types of nanoparticles available here we will focus on liposomes, because these systems have come out as the most clinically successful, given the number of approved formulations and are widely used as drug carriers in oncological clinical setting (Belfiore et al., 2018; Gilabert-Oriol et al., 2018; Lamichhane et al., 2018; Anselmo and Mitragotri, 2019). The reasons of this success are many, such as low toxicity and immunogenicity, easy manufacturing, generally low cost, reproducibility, extreme versatility, biodegradability.

Liposomes are lipid vesicles, in the size range of 50–500 nm, formed by bilayers composed typically by phospholipids and cholesterol, entrapping an aqueous core. Therefore, they have both a hydrophilic region (the aqueous core) and a hydrophobic region (the lipid bilayer) thus being able to load hydrophilic, hydrophobic and amphiphilic payloads and protecting them from degradation in the biological milieu. Drugs can be loaded into liposomes either passively, by adding them during liposome preparation (mixed to lipids, or present in the hydration solution) or actively, by forcing them to cross the lipid bilayer of preformed liposomes in response to a pH gradient across it. This last process, also defined as remote loading, is used for weak alkaline or weak acidic compounds (Madden et al., 1990) and allows highly efficient loading.

As mentioned above a great advantage of liposomes is their extreme flexibility, in fact, since their assembling is controlled by non-covalent interactions, their formulation and surface decoration can be quite easily modulated to the needs. Therefore, in response to the need of prolonging their circulation half-life, polyethylene glycol (PEG) chains were included into the lipid bilayer (Klibanov et al., 1990) thus providing a hydrophilic PEG shell on liposome surface that hinders the aggregation of plasma proteins and delays recognition and clearance by the reticuloendothelial system (RES) (Senior et al., 1991; Allen, 1994; Allen et al., 2002).

A higher target specificity or other specific functions (such as controlled release) can be ascribed to liposomes by surface decoration through ligands or antibodies, surface charge modulation or the inclusion of specific functionalities in the lipid bilayer (Zhao and Rodriguez, 2013). An important duty of liposomes is delivering their payload only to sick districts, thus avoiding healthy tissues and hence side effects. Liposomes can reach sick districts either by passive or active targeting. Passive targeting exploits a combination of the physicochemical feature of liposomes, *i.e.* their stability in the blood stream, and the microanatomy of tumors, *i.e.* their extensive fenestrated vasculature and impaired lymphatic system. In fact, the long circulation time gives liposomes the chance of multiple contacts with the tumor vasculature from where they can extravasate through fenestrations into the tumor microenvironment. On the other hand, active targeting involves recognition of the target by liposomes *via* ligand/receptor or antibody/antigen interaction (Zhao and Rodriguez, 2013).

Here we will review literature acquisitions concerning the use of liposomes to improve the bioavailability of autophagy inhibitors. Once at the target the drug can be release passively or upon a trigger that can originate from the microenvironment, for example the acidic environment of tumor tissues, or by the outside (light, temperature, magnetic field, ultrasounds) (Needham et al., 2000; Alhmady et al., 2012; Chen et al., 2013; Adriyanov et al., 2014; Luo et al., 2016a; Luo et al., 2016b). Passive release does not require further liposome functionalization, whereas in the case of triggered release liposome have to be engineered *ad hoc*. Interesting examples of the use of liposomes to control autophagy concern the treatment of glioma, one of the most common and aggressive brain tumor in adults. Actually, the central nervous system (CNS) is protected by the blood–brain-barrier (BBB), a highly selective border formed by a monolayer of endothelial cells that segregates the CNS from systemic influences while providing the transport of nutrients. The transport barrier includes para- and transcellular transport. The functional peculiarities of endothelial cells of BBB are determined by the unique features of their morphology distinguishing them from other endothelial cells. The absence of cytoplasmic fenestrations and the formation of tight junctions between the membranes of neighboring cells, mediated by transmembrane proteins located within the paracellular space, prevents paracellular transport. The transcellular control is determined by the presence of ABC-transporters, which provide not only the barrier control but also restrict transport of most medicinal drugs to the brain and tumors. Therefore, the main objective in the development of new therapies for the CNS is represented by the development of nanocarriers capable of loading given drugs, crossing the BBB and releasing the drugs into the target districts (Singh et al., 2017; He et al., 2018; Feng et al., 2019). Nanoparticles can cross the BBB by: i) transcellular diffusion (transcytosis), ii) receptor mediated transport, iii) carrier mediated transport (Alam et al., 2010; Alyautdin et al., 2014). Therefore, it is most often necessary to functionalize liposomes to cross the BBB. Another important aspect in the delivery of drugs to the CNS is that liposomes besides protecting their payload from the degradation of the biological environment, protect them also from the action of efflux pumps.

The treatment of glioma with a potent inhibitor of angiogenesis and cancer proliferation was impaired by the induction of autophagy (Shen et al., 2013). As mentioned above, HCQ is known to inhibit autophagy and is capable of sensitizing various tumors to the effects of chemotherapy, however, the nonselective distribution *in vivo* and the low capability to cross the BBB restrict its clinical use as well as the co-delivery of HCQ and ZD6474 in the treatment of glioma (Gustafson et al., 2006; Rangwala et al., 2014; Rosenfeld et al., 2014). Aimed at targeting specifically glioma cells, liposomes functionalized with R6dGR peptide (R6dGR-Lip), that is able to recognize integrin receptors ανβ3 and neuropilin-1 (NRP1), a membrane-bound co-receptor of tyrosine kinase receptor, were developed (Wang et al., 2018). NRP1 is highly expressed in endothelial cells and it is also involved in the vascularization and progression of other types of cancer, such as prostate, breast, colon and lung. Treatment, both *in vitro* and *in vivo*, with ZD6474 and HCQ inhibitors encapsulated in R6dGR-Lip liposomes, lead to block efficiently the autophagic flow and to sensitize glioma cells (Wang et al., 2018).

Recently it was reported about the importance of using ultrasound-targeted microbubble destruction technique (UTMD) to induce a transient and reversible BBB disruption, which greatly simplifies intracerebral drug delivery (Wischhusen and Padilla, 2019). In this technique gas bubbles, activated from ultrasounds cause biophysical shock effects that can permeate biological barriers (Wischhusen and Padilla, 2019). A smart “all-in-one” nanosensitizer platform was developed by uniting sonoactive chlorine c6 (Ce6) and HCQ into angiopep2-peptide-functionalized liposomes (ACHL). ACHL selectively accumulated in the brain tumor due to the transient opening of BBB upon application of UTMD. A second ultrasound stimulation activated the Ce6, triggering the release of HCQ that blocked the degradation of autophagosomes, by increasing oxidative damage, inducing apoptosis and inhibiting mitophagy (Qu et al., 2019).

Another interesting paper reports on the efficacy of multifunctional vinblastine liposomes equipped with transferrin receptor binding peptide TfR-T_12_ and octa-arginine conjugate stearyl-R8 that triggered necrosis, apoptosis and programmed cell death *via* autophagy in brain-glioma-bearing mice (Mu et al., 2017).

The dense stromal structure of pancreatic cancer prevents therapeutic efficacy, leading to an average 5 years survival of patients. Autophagy plays a role in inducing the formation of a dense stroma in the ductal adenocarcinoma of the pancreas. This phenomenon is mediated by cancer-associated fibroblasts that are capable of generating collagen and this, on its turn, hinders targeting of cytotoxic drugs to cells. A multifunctional tandem peptide TH-RGD (TR) consisting of cRGD (a peptide with terminal cysteine) and the pH-sensitive TH peptide, was developed to target tumor cells and cancer associated fibroblasts and used to functionalize liposomes (referred as TR-Lip) to co-load HCQ and paclitaxel (PTX) (Chen et al., 2019). TR-Lip were specifically designed to both target integrin αvb3 and promote cell internalization. In fact, when they meet the acidic tumor environment TR histidine residue protonates, so that liposome surface changes from negative to positive, thus stimulating cell internalization by electrostatic attraction, since the plasma membrane is negatively charged. TR-Lip showed indeed a high penetration capacity and were able to effectively inhibit autophagy and stroma fibrosis in pancreatic cells both *in vitro* and *in vivo*. Rapamycin (Rap), an inducer of autophagy by mTOR inhibition in mammalian cells, showed a potent antitumor activity in a range of solid tumors (Cloughesy et al., 2008; Rouf et al., 2009); however, because of its poor water solubility, its therapeutic application has been very limited. Rap loaded liposomes dispersed in a polymeric network of P407 hydrogel were evaluated *in vitro* and *in vivo*. In particular, two different systems were compared, namely Rap-loaded conventional liposomes (R-CL) and Rap-loaded folate-modified liposomes (R-FL); in both cases the hydrogel polymeric network allowing a controlled release of Rap over time. FL cell uptake was 2-fold higher than that of CL, and folate receptor-mediated endocytosis was proved by a competitive assay with folic acid pretreatment. In orthotopic bladder cancer mouse model, R-FL/P407-treated groups showed enhanced *in vivo* anti-tumor efficacy (Yoon et al., 2019).

It was shown that dihydroartemisinin (DHA), a derivative of artemisinin, increases the efficacy of epirubicin-based treatment of heterogeneous breast cancer by inducing autophagy and apoptosis (Hu et al., 2018). The formulation of DHA and epirubicin in PEGylated liposomes enhanced the anticancer activity in breast cancer-bearing mice. It was suggested that this effect is due to the protracted circulation of drug loaded liposomes in the blood and to the enhanced concentrations of drugs in cancer tissues (Hu et al., 2018). Co-encapsulation of DHA and doxorubicin (DOX) in mannosylated liposomes to target drug-resistant human colon tumor HCT8/ADR cells overexpressing the mannose receptor (Kang et al., 2017) resulted in high accumulation of DOX in the nuclei. Administration of mannosylated liposomes loaded with DHA and DOX to a subcutaneous HCT8/ADR tumor xenograft model, gave a tumor inhibition rate of 88.6%, with respect 47.4% and 70.5% in the treatment with free DOX or free DOX+DHA (Kang et al., 2017), due to nuclear accumulation of the drugs, enhanced cancer cell apoptosis and the induction of autophagy.

A study showed the efficacy of PTX co-encapsulated with CQ in liposomes composed of soybean phosphatidylcholine and cholesterol in PTX-resistant derivatives of human lung adenocarcinoma (A549/T) cells, PTX-resistant derivatives of human ovarian carcinoma (A2780/T) cells, and mouse sarcoma (S180) cells. It was shown that liposomal CQ can sensitize PTX-resistant cell by means of autophagy inhibition and competitively binding with multidrug-resistance transporters. Further, co-encapsulation of PTX and CQ in liposomes resulted more efficient than the mixture of PTX liposome plus CQ liposome. This composite formulation also achieved significantly stronger anticancer efficacy *in vivo* with respect to PTX liposome/CQ liposome mixture (Gao et al., 2015).

Another study demonstrated the efficacy of liposome co-encapsulated DOX and CQ with respect to liposomal DOX or free DOX, both on human breast cancer line multicellular spheroid and on transgenic zebrafish models (Gao et al., 2017).

In malignant melanoma, the relevant level of mortality in patients has to be ascribed to detachment, dissemination, extravasation and colonization of metastases. Autophagy is one of the mechanisms involved in promoting tumor metastasis (Mowers et al., 2017; Mowers et al., 2018). The synergistic antimetastatic and antitumor effects of R8-dGR peptide functionalized liposomes loaded with PTX and HCQ (PTX/HCQ-R8-dGR-Lip) was investigated *in vitro* and *in vivo* (Yin et al., 2018). R8-dGR peptide decoration increased tumor delivery both *in vitro* and *in vivo*. Further, PTX/HCQ-R8-dGR-Lip showed in B16F10 cells, an optimal inhibitory effect on migration, invasion and anoikis resistance. Meanwhile, studies of the antimetastatic mechanism have shown that the combination of PTX and HCQ autophagy inhibitor has suppressed the degradation of paxillin and the expression of MMP9 and MMP2. In addition, HCQ interfered with CXCR4/CXCL12 axis that can promote invasion and metastases of melanoma in an autophagy-independent way.

Esophageal squamous cell carcinoma has been shown not to respond to single drug regimen due to heterogeneous composition of cancerous cells. Also in this situation autophagy inhibitors have been described as effective in overcoming MDR.

5-fluorouracil (5-FU) and autophagy inhibitor LY294002 (LY) loaded PEGylated liposomes were used to target esophageal squamous cell carcinoma. This liposomal formulation showed higher anticancer effect in cancer cells with respect to 5-FU treatment without liposomal formulation. Due to inhibition of autophagy by LY, the enhanced sensitivity of cancer cells to 5-FU was revealed (Feng et al., 2018).

In **Table 1** the peculiar characteristics of the liposomal preparations mentioned above are listed.

**Table 1:** The lipid composition of liposome preparations, the modality of drug loading, liposome size, and the value of liposome surface potential (mV).

Lipid composition	Modality of drug loading	Liposome size (nm)	Liposome surface potential (mV)	EE%	Reference
SPC7chol/DSPE- PEG2000-R6dGR (62:33:5 molar ratio)	ZD6474: passive l HCQ: remote	100	−4.6	70 90	(Wang et al., 2018)
DSPC/DOPC/DSPE-mPEG2000/DSPE-mPEG2000-angiopep-2/chol (55:2:2.5:2.5:38 molar ratio)	Ce6: passive HQC: remote	140	–	54 63	(Qu et al., 2019)
EPC/chol/TfR-T12-PEG2000-DSPE/stearyl-R8 (60:30:3: molar ratio)	Vinblastine: remote	110	−0.97	96	(Mu et al., 2017)
SPC/chol/DSPE- PEG2000-TR (62:30:8 molar ratio)	HCQ: remote PTX: passive	130	−25	81 84	(Chen et al., 2019)
SPC/chol/DSPE-PEG2000-Fol (89.5:10:0.5 molar ratio)	Rap: passive (added at 10% molar ratio)	160	−17	42	(Yoon et al., 2019)
EPC/chol/PEG2000-DSPE (65:30:5 molar ratio)	DHA:passive epirubicin: remote	100	neutral	90	(Hu et al., 2018)
SPC/chol/DSPE- PEG2000-Mannose (27:11:0.6 molar ratio)	DOX: passive DOX/lipid 0.02:10 (w/w) DHA:passive DHA/lipid 1:10 (*w*/*w*)	160	−16	95 95	(Kang et al., 2017)
PTX (4 mg), soybean phos-phatidylcholine (500 mg), and chol (100 mg)	PTX: passive CQ: remote	55	Almost neutral	95 85	(Gao et al., 2015; Gao et al., 2017)
SPC/chol/DSPE- PEG2000- R8-dGR (62:33:5 molar ratio)	PTX: passive HCQ: remote	100	–	85	(Yin et al., 2018)
EPC/DSPE-PEG/chol (10:4:1 molar ratio − total 10 mg)	5-FU: passive (10 mg)LY: passive (1.5 mg)	150	25	93 86	(Feng et al., 2018)

SPC, Sphingosylphosphorylcholine; DSPE, 1,2-distearoyl-sn-glycero-3-phosphoethanolamine; EPC, egg phosphatidyl- choline; chol, cholesterol.

## Conclusions

Autophagy is often altered in tumors, however its role in cell death or survival is controversial. Currently many studies identify in basal autophagy a protective role for the tumor cell, providing nutrients and, therefore, promoting the uncontrolled growth and survival of the cell in the hypoxic areas of the tumor microenvironment.

In conclusion, the critical analysis carried out in this review suggests that the use of liposome as nanovector, in which it is possible to insert both anticancer drugs and products capable of inhibiting the protective autophagic mechanism, may be a viable strategy for improving reactivity or reduce resistance to cancer therapies.

In addition, the data from this review show how important it is to exploit liposomal versatility and non-toxicity together with cutting-edge technology to achieve results that lead increasingly to translation in the clinical field.

## Author Contributions

MC contributed to the drafting of the following chapters: autophagy in cancer, multidrug resistance in cancer and MDR and autophagy. She also proposed and carried out the explanatory schemes in the review. GM carried out a critical analysis on the drafting of the review and in particular on the role of liposomes in drug resistance. SM was responsible for the organization, preparation and final revision of the manuscript, also contributed to the drafting of the chapters: new autophagy inhibitors for cancer treatment and liposomes as an alternative strategy against autophagy-related MDR.

## Funding

Institutional funding support has been used.

## Conflict of Interest

The authors declare that the research was conducted in the absence of any commercial or financial relationships that could be construed as a potential conflict of interest.
